# Folliculin gene-negative Birt-Hogg-Dube syndrome: a case report

**DOI:** 10.1097/MS9.0000000000001496

**Published:** 2024-01-03

**Authors:** Mohammad F. Dwikat, Jehad Azar, Rama Rabayah, Ruba Salameh, Fatima Abdeljaleel, Waseem Almadhoun, Alaa Ayyad, Farah Ibraik, Omar Safarini

**Affiliations:** a Department of Internships, Ministry of Health, Nablus; b Internal Medicine Department, Al-Shifa Medical Complex, Gaza; c Internal Medicine Department, Ibn Sina Specialized Hospital, Jenin; d Internal Medicine Department, Palestine Medical Complex, Ramallah, Palestine; e Internal Medicine Department, Cleveland Clinic Fairview Hospital; f Pulmonary and Critical Care Department, Cleveland Clinic Foundation: Cleveland Clinic, Ohio; g Internal Medicine Department, MedStar Union Memorial Hospital, Maryland, USA

**Keywords:** Birt-Hogg-Dubé syndrome, case report, folliculin gene mutation, pulmonary cystic disease, renal tumor, secondary spontaneous pneumothorax

## Abstract

**Introduction and importance:**

Birt-Hogg-Dube (BHD) is a rare genetic disorder that results from a mutation in the folliculin (FLCN) gene. Manifestations include pulmonary cysts, fibrofolliculomas, renal tumors, and pneumothoraces. Genetic testing can be used to confirm the diagnosis when suspected. BHD syndrome is diagnosed in patients with negative FLCN gene results using diagnostic criteria.

**Case presentation:**

A male in his 20s presented with recurrent pneumothoraces. A physical examination revealed bumps on his face and upper body. A chest computed tomography scan revealed cystic lesions. Blood tests, ESR, and CRP levels were unremarkable. Punch skin biopsy revealed fibrofolliculomas. Genetic testing for the FLCN mutation returned negative. His history, physical exam, imaging, and histopathology suggested BHD syndrome despite having a negative family history and genetic analysis. Eventually, the patient was diagnosed with FLCN gene-negative BHD syndrome.

**Clinical discussion:**

More than a hundred families have been identified to have BHD worldwide. There are a few cases in the literature describing patients phenotypically presenting with BHD despite having a negative genetic analysis. One study in Japan found 16 out of 157 individuals having a clinical presentation of BHD with no mutations. Also, decreased expression of the FLCN mRNA may lead to BHD.

**Conclusion:**

BHD syndrome can present with a negative FLCN gene mutation; however, patients must meet the known diagnostic criteria such as criteria made by Menko *et al*., Gupta *et al*., and Schmidt *et al*. in order to have a diagnosis of BHD syndrome. Also, a qualitative decrease of FLCN with the absence of mutations may also lead to BHD.

## Background

Highlights

- Birt-Hogg-Dube (BHD) is a rare genetic disorder that results from a mutation in the *folliculin* (FLCN) gene.
- BHD syndrome can also have negative gene analysis without the FLCN gene mutation.
- Diagnosis of BHD should rely on the known diagnostic criteria.
- Quantitative loss of FLCN without a gene mutation can also lead to BHD.

Birt-Hogg-Dubé syndrome (BHD) is a rare disorder caused by germline loss of function mutations in the *Folliculin* (FLCN) gene, which encodes the protein FLCN. In the general population, there are around two incidences of BHD for every million people, regardless of gender^1^. If not diagnosed properly, BHD can have devastating effects on patients due to its complications that affect multiple organ systems. Clinical manifestations of BHD can vary significantly, they include cutaneous lesions known as fibrofolliculomas, pulmona ry cysts, pneumothoraces as well as renal tumors. Rarely, patients can also develop renal cysts^2^. On computed tomography (CT), patients usually have several, bilateral, round, irregular, or willow-like pulmonary cysts. Furthermore, CT can also show large cysts (>2 cm) close to the mediastinum, spine, and under the pleura^3^. In contrast, other cystic lung diseases such as lymphocytic interstitial pneumonia (LIP), amyloidosis, and Langerhans cell histiocytosis (PLCH) are associated with pulmonary nodular lesions, which are typically absent in patients with BHD syndrome^4^.

The diagnosis of BHD relies on a combination of clinical evaluation, family history, and genetic testing. Genetic testing for mutations in the *FLCN* gene should be performed when possible since it can confirm over 90% of cases that fulfill the diagnostic criteria for BHD. However, a negative FLCN genetic test does not exclude this syndrome^5^.

Missing the diagnosis of BHD can lead to devastating complications both on the short and long runs. Over 80% of patients having BHD have lung bulla; of which approximately a quarter develop pneumothoracis which can be life-threatening even in children^6^. Also, 14–35% of patients diagnosed with BHD have a risk of developing renal cancer^7^. In patients with a negative FLCN gene, the diagnosis of BHD syndrome is made by the alternative diagnostic criteria (Table 1). There are studies in the literature that describe patients with clinical criteria similar to BHD without any mutations found in genetic analysis. However, to our knowledge, this is the first case report to describe a FLCN-negative BHD patient from presentation to treatment in detail. The authors believe this will contribute to the field by discussing the presentation of BHD and the criteria followed to confirm the diagnosis without necessarily having a positive gene mutation. This will allow physicians to lower their suspicion threshold for BHD even if the genetic analysis turns out to be negative. Therefore, in this paper, we report a male in his 20s who presented with a skin rash and recurrent pneumothoraces and was eventually diagnosed with FLCN gene-negative BHD syndrome. The Surgical CAse REport (SCARE) 2020 criteria were followed while reporting this case study^8^.

**Table 1:** Well-established criteria for diagnosis of Birt-Hogg-Dube syndrome

Diagnostic criteria	Notes	Major criteria		Minor criteria		
Menko and colleagues^5^	Diagnosis is confirmed when one major or two minor criteria are met.	A minimum of five skin manifestations (either trichodiscomas or fibrofolliculomas), with at least one of them diagnosed by histopathological exam and occurred in adulthood		Renal involvement, which should be either early onset renal cancer (i.e., occurred before the age of 50) or multifocal RCC or bilateral RCC, or chromophobe and oncocytic tumor		
		A known mutation in the FLCN gene		Bilateral cysts within the lung bases without a known cause (with or without spontaneous pneumothorax)		
				Family history with a first-degree relative diagnosed with BHD		
Schmidt and Linehan, 2015^19^	Any one of the following four criteria:	A minimum of 2 skin papules clinically consistent with fibrofolliculoma/ trichodiscoma and a minimum of 1 fibrofolliculoma confirmed by biopsy	Multiple bilateral basilar pulmonary cysts +/- a history of spontaneous pneumothorax before the age of 40, a family history of these pulmonary manifestations supports this criterion	Bilateral, hybrid oncocytic tumors OR multifocal chromophobe renal carcinomas a family history of renal tumors or being diagnosed at an age <50 years support this criterion	A combination of these pulmonary, renal, or cutaneous manifestations presenting in the patient or having a family history of these manifestations	
Gupta and colleagues^20^	Any of the following:	Definite Pulmonary BHD (any one of the following four)	Probably Pulmonary BHD (one of the following two)	Possible Pulmonary BHD Compatible or characteristic lung CT	Characteristic lung CT features: Multiple well-defined air-filled cysts with thin walls that are round, elliptical, or lentiform in shape, with basilar, medial, and subpleural distributions predominating, with preserved or increased lung volume and no other significant pulmonary involvement (in particular, exclude interstitial lung disease)	Compatible HRCT findings: Cysts with thin walls lacking the more common elliptical form or subpleural distribution
		Characteristic or compatible lung CT PLUS skin biopsy positive for fibrofolliculoma or trichodiscoma	Characteristic lung CT PLUS exclusion of TSC and LAM PLUS personal or family history of pneumothorax			
		Characteristic or compatible lung CT PLUS family history of BHD in a 1st- or 2nd-degree member				
		Characteristic or compatible lung CT PLUS renal chromophobe adenoma or oncocytoma confirmed by biopsy	Compatible lung CT PLUS exclusion of TSC and LAM PLUS either a family or personal history of renal tumors OR skin angiofibroma OR renal angiomyolipoma			
		Characteristic or compatible lung CT PLUS genetic testing positive for BHD confirmed by biopsy				

BHD, Birt-Hogg-Dube Syndrome; FLCN, folliculin gene; HRCT, high-resolution computed tomography; LAM, lymphangioleiomyomatosis; RCC, renal cell carcinoma; TSC, tuberous sclerosis complex.

In patients with a negative FLCN gene, the diagnosis of BHD syndrome is made by following the proposed alternative diagnostic criteria. Patients must fulfill one major and two minor criteria (Table 1). In this paper, we report a male in his 20s who presented with a skin rash and recurrent pneumothoraces and was eventually diagnosed with FLCN gene-negative BHD syndrome.

## Case presentation

**Figure 1:**
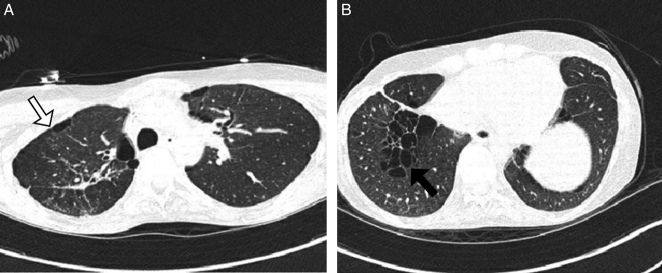
Chest computed tomography (CT) scan images for a male patient with Birt-Hogg-Dube disease: (A) White arrow indicates loss of right lung volume after apical pneumonectomy with compensatory hyperinflation of the left side; (B) Black arrow indicates diffuse irregular shape thin wall cystic changes that are more predominant in the right lower lobe, along with peri-mediastinal and peri-bronchovascular distribution.

**Figure 2:**
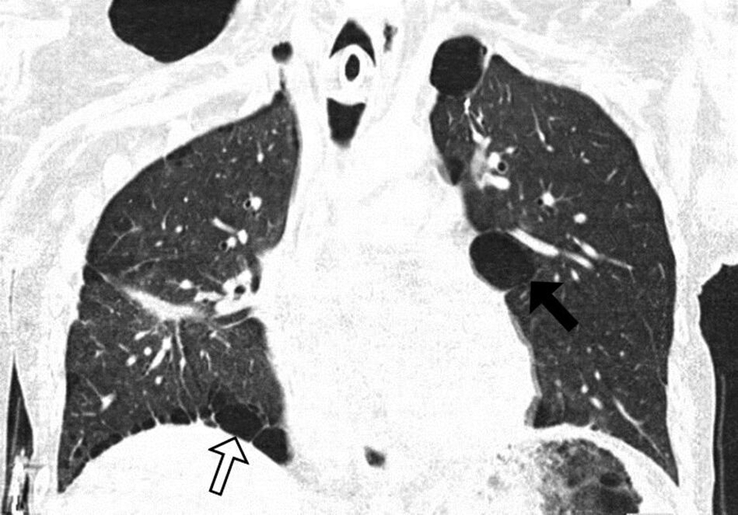
Chest computed tomography scan; coronal view: multiple irregular shape, variable size cystic disease with thin well-defined walls, more pronounced in the right lower lobe, subpleural (white arrow) and peri-mediastinal in location.

A male patient in his 20s presented to our hospital due to recurrent pneumothoraces. Initially, he was hospitalized with a spontaneous right-sided pneumothorax, for which he underwent video-assisted thoracic surgery (VATS), along with apical resection of the right lung and sticking of pleural layers (pleurodesis). Postoperatively, he had an episode of hemothorax, for which he underwent a surgical evacuation. The physical exam upon his initial presentation was only positive for a right-sided pneumothorax. A chest CT scan revealed bilateral pulmonary cystic changes (Figs. 1, 2). An abdominal CT showed no renal abnormalities.

One year later, he developed a spontaneous left sided pneumothorax; VATS was performed, and both subpleural bleb resection and pleurodesis were done. Upon physical examination he was noted to have diffuse small (2–4 mm) flesh-colored bumps of smooth surface and dome shape located on his face and upper trunk; consistent with fibrofolliculomas.

A few months later, the patient developed respiratory failure; complicated by a right-sided hemothorax and complete collapse of the right lung. He underwent VATS again along with the removal of fibrous tissue in the lung (decortication); pulmonary biopsy revealed inflammation of the pleura and subpleural cystic changes, but no other pathological abnormalities were reported. He denied a family history of skin, pulmonary or renal disease; he was a lifetime nonsmoker.

Complete blood count, metabolic panel, ESR, and CRP all were unremarkable. Serum free light chain (FLC) concentration was elevated with a K/L ratio of 1.7 (normal 0.26–1.65). M protein was identified on protein electrophoresis with positive immunofixation. Abdominal fat pad biopsy for suspected amyloidosis, surgical lung biopsy for amyloid and light chain deposition disease, and alpha-1 antitrypsin enzyme all returned negative.

Given his skin lesions, he underwent a punch skin biopsy, which was positive for fibrofolliculoma. Whole-exome gene sequencing, including genetic screening for the FLCN mutation returned back negative. He also had negative testing for Ehlers-Danlos Syndrome mutations as well as mitochondrial gene sequencing.

His history of recurrent pneumothorax, consistent chest CT findings, and fibrofolliculoma on skin biopsy raised suspicion for BHD syndrome; yet his negative family history, absence of renal tumors, and negative genetic screening for the FLCN mutation made the diagnosis challenging. Thus, other diffuse cystic lung diseases (DCLDs) were considered in the differential diagnosis including lymphangioleiomyomatosis (LAM), although the patient’s clinical presentation and gender were atypical for such disease. His serum vascular endothelial growth factor (VEGF) level was normal and HMB-45 staining on lung biopsy was negative. The chest CT findings were also atypical for LIP as well as PLCH. In addition, there were no clinical signs suggesting neurofibromatosis, Marfan, or Ehlers-Danlos syndromes. With the given findings, the patient was diagnosed with FLCN gene-negative BHD syndrome based on the diagnostic criteria illustrated in this paper (Table 1).

The patient had pleurodesis due to his many recurrences of penumothoracis. He did not develop pneumothorax after the pleurodesis was done. In addition, the abdominal imaging did not reveal a malignancy in the kidneys. However, the patient was advised to have renal imaging done every 5 years to screen for renal malignancy.

## Discussion

Some studies indicate that rare diseases affect up to 10% of people worldwide. Most rare diseases are severe, chronic, and even life-threatening^9^. Rare diseases, which are often inherited, frequently present in childhood and can have deleterious long-term effects. Patients with rare diseases often face diagnostic delays; it can take 7 years or more to reach an accurate diagnosis^10^. Inaccurate or delayed diagnoses can make it more difficult to provide treatment and prevent complications of these rare diseases.

BHD syndrome is a rare disease that has an autosomal dominant inheritance. This disorder is characterized by benign skin hamartomas on the head and neck; pulmonary cysts, spontaneous pneumothoraces, and an increased risk of renal cancer^11^. The incidence of BHD syndrome is still unknown but more than a hundred families have been identified worldwide^12^.

Even though this patient’s sex and clinical presentation were abnormal for LAM, other diffuse cystic lung diseases (DCLDs) were taken into consideration in the differential diagnosis. His lung biopsy’s HMB-45 staining came back negative, and his serum VEGF level was within normal limits. In addition, chest CT can be used to differentiate BHD from LAM, PLCH, and LIP^13,14^. In this patient, CT turned out inconsistent with them. Furthermore, no clinical indications of neurofibromatosis, Marfan, or Ehlers-Danlos syndromes were seen.

For long-term follow-up of BHD patients. Due to patients with BHD being at a high lifetime risk of developing RCC^7^, it is recommended that a CT scan of the abdomen with IV contrast or an MRI is conducted at least once every 36 months^15^. However, because MRI produces high-resolution pictures without exposing patients to the cumulative radiation dose of serial CT imaging, it is the preferred method of imaging.

A Japanese study published in 2016^16^ demonstrated that 16 out of 157 individuals suspected to have BHD did not have sequence variants in FLCN coding regions. Additional quantitative PCR tests yielded no duplication/deletion. Regarding the genotype-phenotype correlation of BHD, a German study published in 2018 analyzed possible genotype-phenotype-correlations that might affect RCC penetrance in BHD syndrome patients^7^. Eighty-three patients and their families underwent germline *FLCN* testing. One of the families clinically diagnosed with typical BHD syndrome had no detectable *FLCN* mutation.

A recently-published case report^17^ documented the presence of phenotypical BHD manifestations (skin changes and bilateral renal cysts) in a female patient in her 50s. The patient’s family history was concordant with BHD syndrome with a daughter having recurrent spontaneous pneumothoraces and bilateral renal cysts as well as a brother having bilateral renal cysts. Genetic mutation screens for FLCN and nine other genes were all negative by targeted next-generation sequencing and diagnosis of BHD syndrome was made based on the diagnostic criteria described in this study (Fig. 1).

In addition, Enomoto *et al.* ^18^ reported a case of a 38-year-old man who presented with a left renal tumor and papular facial lesions consistent with fibrofolliculomas; enhanced CT revealed a mass in the left kidney and multiple small basal pulmonary cysts. The patient had a positive family history of skin manifestations and his clinical picture was suggestive of BHD syndrome. The FLCN genetic testing of the patient and his family revealed a large genomic deletion including the promoter region of FLCN exon 1. Surprisingly, the mRNA quantity was half compared to healthy volunteers. RCC cells did not show any mRNA transcripts. With the immunohistochemical examination confirming the absence of FLCN expression. This may show that a low quantity of the protein in the absence of a mutation can also lead to BHD syndrome.

### Criteria for BHD syndrome

The most widely used diagnostic criteria for BHD has been proposed by Menko and colleagues and consists of the following^5^, this criteria along with other valuable criteria are presented in Table 1 ^19,20^.

Patients should meet either one major criterion or two minor criteria to be diagnosed with BHD.

Major criteria include: (i) a minimum of five skin manifestations (either trichodiscomas or fibrofolliculomas), with at least one of them occuring in adulthood and diagnosed by histopathological exam or (ii) a known mutation in the FLCN gene. While the minor criteria include: (i) bilateral cysts within the lung bases without a known cause (with or without spontaneous pneumothorax), (ii) renal involvement, which should be either early onset renal cancer (i.e. occurred before the age of 50) or multifocal RCC or bilateral RCC, or chromophobe and oncocytic tumor, or (iii) family history with a first-degree relative diagnosed with BHD.

Additional diagnostic criteria have been proposed by Gupta *et al*.^20^. These suggested criteria by Gupta and his colleagues re-emphasized the importance of the chest and abdomen CT, skin biopsy, FLCN gene mutation and family history of BHD in making the diagnosis of this rare disease. Moreover, they proposed new diagnostic criteria with confidence level based on the number of positive findings patients might have, which ultimately make the diagnosis of BHD syndrome as definite, probable, or possible.

## Conclusion

- BHD syndrome is an autosomal dominant ​disease with genetic penetrance. BHD syndrome is confirmed by genetic testing.
- Negative genetic testing cannot rule out the disease.
- Certain algorithms are to be followed when there is high suspicion of BHD but genetic testing turns out to be negative.
- Patients are advised to do a CT scan with IV contrast or MRI starting by the second decade of life and at least once every 36 months to exclude RCC.
- The clinical picture (e.g. recurrent pneumothoracis, renal tumors, and fibrofolliculomas) should keep the physician’s suspicion high for the diagnosis of BHD even if genetic testing is negative.

## Ethical approval

Not applicable.

## Consent

Written informed consent for publication of this case and any accompanying images was obtained from the patient. A copy of the written consent is available for review by the Editor-in-Chief of this journal.

## Sources of funding

This case study received no fund from any governmental, private, or nonprofit funding organizations.

## Author contribution

M.F.D. and J.A.: contributed equally to the work and should be considered co-first authors; M.F.D. and J.A.: contributed to concept and design of the study. All authors contributed to interpretation and assembly of data and media, drafting or revising the article, gave final approval of the manuscript, and agreed to be accountable for all aspects of the work.

## Conflicts of interests disclosures

All authors declare no possible conflicts of interest with regard to any part of this research.

## Research registration unique identifying number (UIN)

1. Name of the registry: not applicable.
2. Unique identifying number or registration ID: not applicable.
3. Hyperlink to your specific registration (must be publicly accessible and will be checked): not applicable.

## Guarantor

Dr Mohammad F. Dwikat.

## Data availability statement

No datasets have been generated and/or analyzed in this article.

## Provenance and peer review

Not commissioned, externally peer-reviewed.
